# Paroxysmal exercise‐induced dyskinesia without involuntary movements

**DOI:** 10.1002/jgf2.438

**Published:** 2021-03-24

**Authors:** Kosuke Ishizuka, Tomoko Tsukamoto, Masatomi Ikusaka

**Affiliations:** ^1^ Department of General Medicine Chiba University Hospital Japan

**Keywords:** involuntary movements, paroxysmal dyskinesias, paroxysmal exercise‐induced dyskinesia

## Abstract

A 66‐year‐old British man was referred to our department because of a 2.5 year history of bilateral paroxysmal weakness of the lower limbs. It occurred when he walked for about 10 minutes, so he would stop in place and spontaneously rest for up to 15 minutes. When carbamazepine 200 mg/day was administered, the severity and frequency of the symptoms reduced by half and resolved when the dose was increased to 300 mg/day. Even if no involuntary movement is observed, paroxysmal exercise‐induced dyskinesia should be considered in patients with paroxysmal painless gait disturbance, and a therapeutic trial of anticonvulsants may be helpful.

## Graphical abstract

Paroxysmal dyskinesias are a group of movement disorders characterized by attacks of hyperkinesia, which include combinations of dystonia, choreoathetosis, and ballism with intact consciousness. There are different subtypes of paroxysmal dyskinesias that include paroxysmal exercise‐induced dyskinesia (PED), paroxysmal kinesigenic dyskinesia (PKD), paroxysmal non‐kinesigenic dyskinesia (PNKD), and paroxysmal hypnogenic dyskinesia (PHD). Even if no involuntary movement is observed, paroxysmal exercise‐induced dyskinesia should be considered in patients with paroxysmal painless gait disturbance, and a therapeutic trial of anticonvulsants may be helpful.

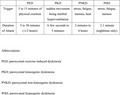

## 1 INTRODUCTION

Paroxysmal dyskinesias are a group of movement disorders characterized by attacks of hyperkinesia with intact consciousness.^1^, ^2^ Paroxysmal dyskinesias are rare disorders and may be misdiagnosed as epilepsy or psychogenic disease.^3^ The clinical course of this case is valuable for the common knowledge of paroxysmal dyskinesias, and we report this case with a review of the literature.

## 2 CASE PRESENTATION

A 66‐year‐old British man was referred to our department because of a 2.5 year history of bilateral paroxysmal weakness of the lower limbs that occurred while walking. This occurred frequently, so he visited several medical facilities, including some in the UK. However, brain magnetic resonance imaging (MRI), brain magnetic resonance angiography (MRA), spinal MRI, myelogram, lower extremity angiography computed tomography, electromyography, muscle biopsy, and electroencephalogram were normal. Because the cause of his symptoms was unknown, he was referred to our department. Paroxysmal lower limb weakness occurred when he walked for about 10 minutes, during which both knees trembled and lost strength, so he would stop in place and spontaneously rest for up to 15 minutes. He had never fallen due to symptoms. Although his symptom resembled intermittent claudication, he did not have any pain and could sometimes walk through a long distance when this attack did not happen. His symptoms did not interfere with his work as an English teacher. There was no history of rapid eye movement sleep behavior disorder, autonomic symptoms such as constipation and orthostatic hypotension, hyposmia, bradykinesia, tremor, hypophonia, gait changes, dysphonia, dysarthria, decreased facial expression, depression, or anxiety. Past medical history was only remarkable for gastroesophageal reflux disease, for which he was on 20 mg/day of oral esomeprazole. His family history was unremarkable. He reported a smoking history of 10 cigarettes per day for over 50 years and social/opportunity drinking. Physical examination was unremarkable. Neurological examination showed intact higher functions and normal cranial nerves, muscle tone, power, and reflexes. There was no bradykinesia, rigidity, tremor, hypophonia, micrographia, decreased arm swing, or short step length. There was no postural reflex disorder, and Myerson's sign was negative. Laboratory tests did not show any abnormalities.

Involuntary movements should be considered as part of the differential diagnosis when there is paroxysmal weakness in the absence of kneeling or falling. The symptoms were induced during a 10 minute test walk performed in an outpatient space, and although no clear involuntary movements could be observed, we considered paroxysmal exercise‐induced dyskinesia which is one of the paroxysmal dyskinesias, because the attacks were precipitated by about 10 minutes of physical exertion and not by sudden movement, and the attacks lasted up to 15 minutes. Four weeks after presentation, carbamazepine 200 mg/day was administered for therapeutic diagnostic purposes for 4 weeks, and the severity and frequency of the symptoms were reduced by half. Eight weeks after presentation, the dose of carbamazepine was increased to 300 mg/day, and the symptoms resolved the next day. Twenty‐one weeks after presentation, in order to differentiate psychogenic diseases such as depression and panic disorder, the oral medication was switched from carbamazepine 300 mg/day to duloxetine 20 mg/day; however, the symptoms returned 2 days later. Twenty‐two weeks after presentation, his oral medication was reverted to carbamazepine 300 mg/day, and his condition improved the following day. Therefore, it was determined that carbamazepine had a specific effect on the primary disease.

## 3 DISCUSSION

Paroxysmal dyskinesias are a group of movement disorders characterized by attacks of hyperkinesia, which include combinations of dystonia, choreoathetosis, and ballism with intact consciousness.^1^, ^2^ The age of onset is usually between childhood and adolescence, but adult‐onset cases have been reported.^1^ Most cases of paroxysmal dyskinesias are sporadic and secondary, whereas familial paroxysmal dyskinesias are inherited in an autosomal dominant manner.^1^ There are different subtypes of paroxysmal dyskinesias that include paroxysmal exercise‐induced dyskinesia (PED), paroxysmal kinesigenic dyskinesia (PKD), paroxysmal nonkinesigenic dyskinesia (PNKD), and paroxysmal hypnogenic dyskinesia (PHD) (Table 1).^1^, ^2^, ^3^, ^4^ Paroxysmal kinesigenic dyskinesia is defined as attacks of dyskinesia precipitated primarily by sudden movement and typically lasting <5 minutes, whereas, as in this case, PED is characterized by attacks of dyskinesia precipitated by 5‐15 minutes of physical exertion, such as walking and running, and typically lasting for 15‐30 minutes.^1^ Paroxysmal dyskinesia occurs at various sites such as the upper and lower extremities, the face, and the neck, though it occurs more frequently in the lower extremities (75%).^5^ Of the reviewed literature, 48% of paroxysmal dyskinesia cases were unilateral (36% unilateral/one side and 12% unilateral/either side), 35% were bilateral, and 18% were both unilateral and bilateral.^2^ Some cases have also been reported in which involuntary movements are not clear.^2^, ^3^ Because paroxysmal dyskinesia can occur secondary to cerebrovascular disease, brain tumors, and Parkinson's syndrome, neuroimaging (preferably MRI) is important to rule out these etiologies.^1^, ^2^ It is clinically important to differentiate paroxysmal dyskinesia because it can be improved by anticonvulsants.^1^ Paroxysmal dyskinesia is classified as an action dystonia because it is induced by voluntary movement. Paroxysmal exercise‐induced dyskinesia is distinguished from task‐specific dystonia, such as spasticity, which is also an action dystonia, because attacks persist beyond the period of voluntary movement, and movement is not always accompanied by attacks even with triggers.

**TABLE 1:** Clinical characteristics of paroxysmal dyskinesias^1^, ^2^, ^3^, ^4^

	PED	PKD	PNKD	PHD
Trigger	5‐15 min of physical exertion	Sudden movement being startled hyperventilation	Stress, fatigue, menses, heat	Stress, fatigue, menses
Duration of attack	5‐30 min (≦2 h)	A few seconds to 5 min	2 min to 4 h	≦1 min (nighttime only)

Abbreviations: PED, paroxysmal exercise‐induced dyskinesia; PHD, paroxysmal hypnogenic dyskinesia; PKD, paroxysmal kinesigenic dyskinesia; PNKD, paroxysmal non‐kinesigenic dyskinesia.

This case study has several limitations. First, although his symptom is mainly an organic cause as an apparent effect of anticonvulsant that is not due to placebo effect, superimposed psychosomatic cause was not completely ruled out because we did not wait an adequate length of time before evaluating the effect of selective serotonin reuptake inhibitors. Second, PED is reported to be a presenting feature of patients with early‐onset Parkinson's disease.^6^ Therefore, in order to establish an accurate diagnosis, despite the absence of a history or physical findings suggestive of Parkinson's disease, we should have considered performing chromosomal testing and a dopamine transporter scan to rule out Parkinson's disease.

## 4 CONCLUSION

Even if no involuntary movement is observed, paroxysmal exercise‐induced dyskinesia should be considered in patients with paroxysmal painless gait disturbance, and a therapeutic trial of anticonvulsants may be helpful.

## CONFLICT OF INTEREST

The authors have stated explicitly that there are no conflicts of interest in connection with this article.

## AUTHOR CONTRIBUTIONS

All authors had access to the data and a role in writing the manuscript.

## INFORMED CONSENT

The patient's written consent was obtained for publication.
